# Do delta waves in the receiver’s brain explain slow tempos of animal communication signals?

**DOI:** 10.1371/journal.pbio.3003814

**Published:** 2026-06-10

**Authors:** Mingzi Xu, Lata Kalra

**Affiliations:** Department of Ecology, Evolution and Behavior, University of Minnesota-Twin Cities, St Paul, Minnesota, United States of America

## Abstract

How does the tempo of animal communication signals evolve? This Primer explores two recent reports published in PLOS Biology, identifying a hotspot of signal tempos between 0.5-4 Hz and independently hypothesizing that delta waves in the receivers’ brains may drive the evolution of this pattern.

## Teaser

How does the tempo of animal communication signals evolve? Two recent reports published in PLOS Biology identified a hotspot of signal tempos between 0.5-4 Hz and independently hypothesized that delta waves in the receivers’ brains may drive the evolution of this pattern.

Like humans, other animals routinely communicate. Signals used in communication are diverse and elaborate. What drives the evolution of these signals remains a central question in evolutionary biology, for which numerous hypotheses have been proposed [1,2]. In the 1990s, a group of related ideas emerged, offering an explanation from the receiver’s side [3–5]. The central philosophy is that neurological biases in the sensory and/or central processing systems of receivers represent a key selective pressure shaping the evolution of signals. An iconic example comes from the African cichlid fishes. As water depth increases, the ambient spectrum becomes redder. Accordingly, color sensitivity of cichlids shows a concerted red shift, which in turn drives the body coloration of males to shift towards red [6]. In other cases, such as some swordtail fishes, the receiver bias lies in not the sensory but the central processing systems [3]. Although this idea was first proposed in the context of sexual selection, the principle transcends communication contexts. Over three decades after the birth of this hypothesis, evidence has mounted [7], but the neurological mechanisms generating the biases, especially those in central processing, remain less well understood.

**Fig 1:**
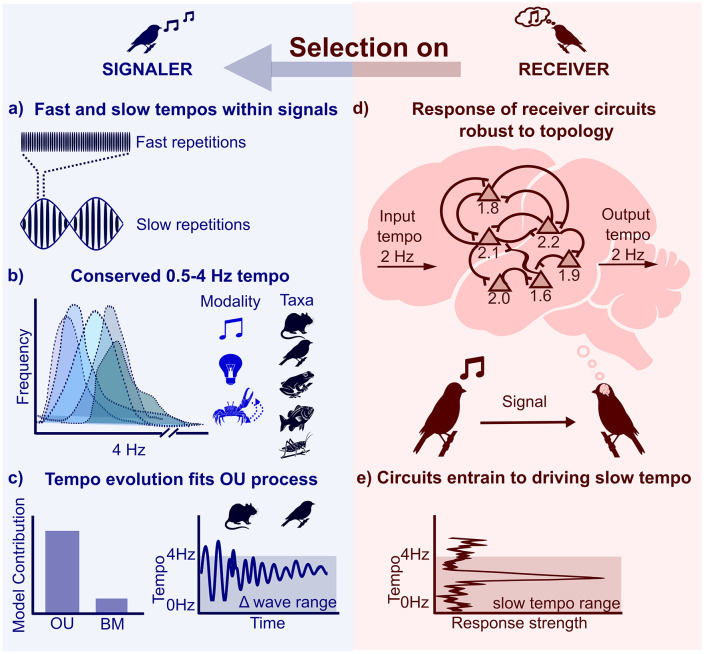
Slow tempos within signals are shaped by neurological biases in receivers’ brains. **(a)** Schematic illustration of fast and slow tempos, shown as the rate at which individual elements were repeated and the amplitude envelope that parses individual elements, respectively, within animal signals. **(b)** Illustrative density plot for signal tempos across taxa and signal modalities. Individual curves represent different taxa. Data from Amichay et al. [8] and Piette et al. [9] converged onto the finding that slow tempos in signals show a conserved local concentration below 4 Hz. The musical note, light bulb, and fiddler crab represent acoustic, bioluminescent, and motion-based signals, respectively. Taxonomic groups are illustrated using silhouettes of mouse, bird, frog, fish, and cricket, respectively. **(c)** Illustrative figures depicting main findings from Piette et al. [9]. Left panel: bar plot showing the Ornstein–Uhlenbeck (OU) model outperforming the Brownian Motion model in a Bayesian framework, signified by higher relative contribution of OU model to the best fit model, suggesting potential stabilizing selection on the conserved slow signal tempos; Right panel: illustrative figure showing convergence to an optimal tempo within the delta wave range under the OU model in mammals and birds. **(d)** Illustration of response by an example neurocircuit consisting of slow oscillators within a receiver’s brain from Amichay et al. [8]. Data showed that circuit response is robust to its topology (i.e., the wiring of neurons). Neurons and circuit topology are represented by triangles and bifurcating lines, respectively. The numeric value below each triangle represents the intrinsic oscillation frequency of the neuron. **(e)** Illustrative plot depicting entrainment of an example small circuit to a driving tempo within the delta band from a neuro-computational experiment by Amichay and colleagues [8].

Many animal signals are temporally repetitive: the songs of birds, frogs, and insects, the flashing of fireflies, and the waving of the major chelae of fiddler crabs all contain elements repeated at relatively regular tempos (Fig 1a). Two recent Discovery Reports in PLOS Biology from Amichay and colleagues [8] and Piette and colleagues [9] focused on the tempo of these signals. Inspired by cricket singing and firefly flashing, and after surveying publications and databases, Amichay and colleagues identified a hotspot of tempos in communication signals between 0.5 and 4 Hz across modalities and taxonomic groups (Fig 1b) [8]. Independently, by analyzing existing recordings, Piette and colleagues demonstrated a similar concentration of slow tempos below 4 Hz among acoustic signals (Fig 1b) [9]. These convergent findings raise an intriguing question: what could have driven the evolution of such a conserved pattern? Amichay and colleagues [8] and Piette and colleagues [9] approached this question from the perspective of computational neurobiology and comparative phylogeny, respectively. In doing so, the two studies have independently arrived at a similar hypothesis: biases arising from slow oscillators or circuits in the receiver’s brain may, at least in part, drive the evolution of signals with slow repetition tempos (Fig 1).

In a comparative phylogenetic study published in the current issue, Piette and colleagues [9] tested the hypothesis that species-level factors including body weight, whether food is chewed, and living environment explain the observed pattern. Phylogenetically controlled Bayesian models failed to reject the null, suggesting that these species-level factors are unlikely to be major contributors to the observed pattern. The authors subsequently examined the dynamics of signal tempo evolution across a phylogeny containing 58 birds and 28 mammals. They tested two alternative hypotheses: that below 20 Hz, signal tempos have evolved randomly, approximated by a Brownian motion model, or that signal tempos have evolved toward an optimum, approximated by an Ornstein–Uhlenbeck (OU) model. Results supported an OU process, with an estimated optimum signal tempo at 2.7 Hz, providing evidence for stabilizing selection for the conserved slow tempos (Fig 1c). The robustness of this conclusion could be improved by increasing both sample size and species representation in future research. The authors hypothesized that the evolution of this pattern could be driven by delta oscillations in the receivers’ brains, a deeply conserved brain wave that matches the observed tempo pattern and has been implicated in sensory processing [10–12].

In another Discovery Report published earlier, Amichay and colleagues proposed a similar hypothesis that the hotspot of slow signal tempos may be an adaptation to the behaviors of small circuits in the receivers’ brains [8]. Is there any evidence that such a mechanism could be plausible? Amichay and colleagues [8] provided an initial test in two computational experiments based on simulated circuits comprising five oscillators. In the first experiment, the authors asked whether the emergent circuit response to a driving signal of 2 Hz depends on circuit topology (i.e., how neurons are connected within a circuit). Results indicated little dependence (Fig 1d). In the second experiment, the authors asked whether the circuit can selectively entrain to a forcing tempo in the delta band. Results showed that entrainment was the strongest when the forcing tempo matched the intrinsic tempo of the circuit itself but not its harmonics, providing initial evidence that circuits consisting of delta-band oscillators can selectively respond to external tempo inputs in the 0.5–4 Hz range (Fig 1e). Additionally, by increasing variability in intrinsic tempo of the neurons and strength of coupling to the driving signal, the circuit can respond to a slightly broader range of forcing tempos. Such flexibility and the robustness to circuit topology may both facilitate evolution and persistence of this mechanism throughout deep evolutionary history. This work using small, simple circuits offers an early proof-of-concept support for the hypothesis. As the authors noted, more complex computational models are needed to obtain sophisticated insights. Meanwhile, identifying a system where this idea can be tested empirically would be a challenging but fruitful endeavor.

Taken together, these two studies converged on a similar idea and support an intriguing hypothesis that biases in the receiver’s brain may, at least in part, drive the evolution of signals with slow repetition tempos [8,9]. This hypothesis transcends signal modalities and taxa and proposes a neuronal mechanism for how conserved biases in central processing of receivers can emerge (Fig 1).

These two studies are an excellent example showcasing the value of integrating evolutionary thinking into neurobiological research. Signals with slow tempos have long been recognized in behavioral ecology, and delta oscillations were discovered in neurobiology over a century ago. Yet only recently with increased cross-disciplinary integration has a potential connection between these phenomena begun to emerge. Together, these two studies help pave the way toward an integrative framework and underscore the importance of collaboration across discipline boundaries as a promising approach for ultimately explaining diverse animal signals.

## Abbreviation:

OU: Ornstein–Uhlenbeck

## References

[1] Forrest TG. From sender to receiver: propagation and environmental effects on acoustic signals. Am Zool. 1994;34(6):644–54. doi: 10.1093/icb/34.6.644

[2] Podos J. A performance constraint on the evolution of trilled vocalizations in a songbird family (passeriformes: emberizidae). Evolution. 1997;51(2):537–51. doi: 10.1111/j.1558-5646.1997.tb02441.x 28565357

[3] Basolo AL. Female preference for male sword length in the green swordtail, *Xiphophorus helleri* (Pisces: Poeciliidae). Anim Behav. 1990;40(2):332–8. doi: 10.1016/s0003-3472(05)80928-5

[4] Endler JA. Signals, signal conditions, and the direction of evolution. Am Nat. 1992;139:S125–53. doi: 10.1086/285308

[5] Ryan MJ, Rand AS. The sensory basis of sexual selection for complex calls in the túngara frog, *Physalaemus pustulosus* (sexual selection for sensory exploitation). Evolution. 1990;44(2):305–14. doi: 10.1111/j.1558-5646.1990.tb05200.x 28564368

[6] Seehausen O, Terai Y, Magalhaes IS, Carleton KL, Mrosso HDJ, Miyagi R, et al. Speciation through sensory drive in cichlid fish. Nature. 2008;455(7213):620–6. doi: 10.1038/nature07285 18833272

[7] Cummings ME, Endler JA, Handling editor: Rebecca C Fuller. 25 Years of sensory drive: the evidence and its watery bias. Curr Zool. 2018;64(4):471–84. doi: 10.1093/cz/zoy043 30108628 PMC6084598

[8] Amichay G, Balasubramanian V, Abrams DM. A widespread animal communication tempo may resonate with the receiver’s brain. PLoS Biol. 2026;24(4):e3003735. doi: 10.1371/journal.pbio.3003735 41980041 PMC13078620

[9] Piette T, Cathcart C, Barbieri C, Ming KM, Grandjean D, Bickel B, et al. Animal acoustic communication has a conserved optimal rhythm within the neural delta range. PLoS Biol. 2026;24(6):e3003798. 10.1371/journal.pbio.3003798 PMC13249164 42263115

[10] Morillon B, Arnal LH, Schroeder CE, Keitel A. Prominence of delta oscillatory rhythms in the motor cortex and their relevance for auditory and speech perception. Neurosci Biobehav Rev. 2019;107:136–42. doi: 10.1016/j.neubiorev.2019.09.012 31518638

[11] Schroeder CE, Lakatos P. Low-frequency neuronal oscillations as instruments of sensory selection. Trends Neurosci. 2009;32(1):9–18. doi: 10.1016/j.tins.2008.09.012 19012975 PMC2990947

[12] Senkowski D, Engel AK. Multi-timescale neural dynamics for multisensory integration. Nat Rev Neurosci. 2024;25(9):625–42. doi: 10.1038/s41583-024-00845-7 39090214

